# Effect of emotional intelligence on problematic social media use among Chinese college students: mediating role of social exclusion and experiential avoidance

**DOI:** 10.3389/fpsyt.2025.1567060

**Published:** 2025-06-03

**Authors:** Hexu Guan, Sifan Peng, Zixin Liu, Huanran Sun, Hongxuan Wu, Xumei Yao, Zi Chen, Xi Yang

**Affiliations:** School of Psychology, Chengdu Medical College, Chengdu, Sichuan, China

**Keywords:** emotional intelligence, social exclusion, experiential avoidance, problematic social media use, internet addiction, college students

## Abstract

**Objective:**

To examine the mediating effects of social exclusion and experiential avoidance on college students’ emotional intelligence and problematic social media use.

**Methods:**

Using convenience sampling, 1,448 students enrolled at nine public universities in Chengdu, Beijing, Shanghai, and Kunming were recruited from May 1, 2021, to October 28, 2021. The Emotional Intelligence Scale, the Social Exclusion Questionnaire for College Students, the Acceptance and Action Questionnaire, and the Problematic Mobile Social Media Use Assessment Questionnaire for Adolescents were used to conduct the survey.

**Results:**

The results showed that college students’ emotional intelligence was negatively correlated with social exclusion, experiential avoidance, and problematic social media use (*p* < 0.01). Social exclusion among college students was positively correlated with experiential avoidance and problematic social media use (*p* < 0.01), and experiential avoidance was positively correlated with problematic social media use (*p* < 0.01). This study revealed that college students’ emotional intelligence directly influences their problematic social media use. Social exclusion and experiential avoidance mediated, and sequentially chain-mediated, the effects of emotional intelligence on problematic social media use.

**Conclusion:**

Emotional intelligence can potentially influence problematic social media use directly and indirectly through social exclusion and experiential avoidance.

## 1 Introduction

With the rapid development and popularization of the Internet, global Internet usage has increased by 1,331.9% since the beginning of the new century (1). The 53rd Statistical Report on Internet Development in China states that as of December 2023, the Internet penetration rate will reach 77.5%, and the per capita weekly Internet access time will be 26.1 hours. The number of social media users, represented by mobile phone users, reached 1.091 billion (2). Students are heavy social media users; 99.39% of college students use social media daily, 33% use it for 4–6 h a day, and 22.63% use it for more than 7 h per day (3). Compared with offline socialization, the anonymity, idealized self-presentation, lack of time, and geographic constraints that characterize online socialization have lowered the Internet social media use threshold, attracting many college students (4).

Social media significantly strengthens interpersonal communication, maintains social relationships, and alleviates anxiety (5). However, when individuals use online social media with high frequency and intensity to fulfill their psychological needs, it may lead to problematic or pathological use (6, 7). This includes spending most of the day chatting on social media (e.g., WeChat and QQ) and staying up late using TikTok, Weibo, and other social networking services (8), which can lead to addiction-like symptoms (9). This unpleasant condition, known as problematic social media use (PSMU), involves the prolonged and intense use of online social media, which negatively affects the individual’s physical, psychological, and behavioral aspects. However, it does not meet the Diagnostic and Statistical Manual of Mental Disorders’ diagnostic criteria for mental illness (10). According to Jiang, PSMU includes five dimensions: increased viscosity, physiological damage, omission anxiety, cognitive failure, and guilt (11). Among them, “increased stickiness” means that individuals unconsciously use social apps frequently, gradually extend their use time, and become dependent on them, making it difficult for them to control themselves. “Physical damage” refers to the frequent and prolonged use of mobile social networks, which leads to physical problems such as eye fatigue, cervical spine pain, finger discomfort, vision loss, and poor sleep quality. “Missing anxiety” refers to the frequent checking of cell phones for fear of missing important information in social networks, accompanied by feelings of anxiety and uncontrollable urges to use the phone. “Cognitive failure” refers to the frequent and prolonged use of mobile social networks, which leads to distraction, memory loss, and weakening of the ability to think deeply and communicate realistically. “Guilt” refers to the emotional experience of regret and guilt due to the delay in study or work because of spending too much time on mobile social networks (11). PSMU is a subtype of Internet addiction based on traditional Internet addiction. Despite not reaching a pathological level, problematic social media use behaviors can impair an individual’s psychosocial functioning, resulting in depression (12) and anxiety (13); physiological problems such as impaired sleep quality (14, 15) and eating disorders (16); and cognitive impairments such as memory loss and attention deficits (17). This negatively affects physical and mental health. As college students comprise a large percentage of social media users, research has concentrated on the protective and risk factors for PSMU.

Currently, most research on PSMU is similar to that on Internet addiction, with many referencing the interaction of Person–Affect–Cognition–Execution (I-PACE) model of addictive behaviors. This model is the primary theoretical framework for explaining the formation mechanisms of internet addiction, including PSMU (18, 19). This describes how combinations of different factors can lead to Internet-related problems and suggests that core personal characteristics interact with specific affective and cognitive responses, leading to problematic usage patterns. Additionally, according to compensatory Internet use theory (CIUT) (20), individuals use online applications to fulfill their unmet real-life needs. This theory complements the I-PACE model and explains the different factors and motivations that influence PSMU. According to the I-PACE model, emotional intelligence (EI) is one of the core personal traits in the formation and development of PSMU in individuals (21). Previous studies have shown that individuals’ EI is related to social exclusion and that individuals with high social exclusion are prone to compensating through online social media. Due to China’s special accommodation system, all college students must live in school-designated dormitories during the school year. Thus, Chinese college students spend most of their time in group life, they are more sensitive to bad interpersonal relationships and social exclusion, and avoidance behaviors such as interpersonal avoidance increase accordingly. Thus, the sense of social exclusion as an affective factor may be one of the important factors in elucidating the relationship between an individual’s core characteristics (e.g., EI) and coping patterns (e.g., PSMU). According to the I-PACE model, cognitive processes and decision-making coping are among the influences on the formation and development of PSMU in individuals. Chinese college students are more competitive in terms of academics and pressure, which can easily trigger nonadaptive cognitions and avoidance behaviors after being frustrated. Thus, experiential avoidance, as a nonadaptive cognition, can explain to some extent why maladaptive individuals choose to cope with negative events and emotions by overusing online social media.

### 1.1 Emotional intelligence and problematic social media use

Certain core personal traits may be protective of or susceptible to PSMU (18, 19). As a core personal trait, emotional intelligence may be a distal factor influencing PSMU, necessitating its inclusion in various studies.

EI is the capacity to identify, comprehend, control, and use emotions. It encompasses self-emotional assessment, others’ emotional assessment, control, and use (22). The theory of compensatory network use states that adolescents with lower levels of EI who struggle to control and manage negative emotions may turn to social networks to distract themselves and avoid unpleasant emotions. Over time, this coping mechanism may lead to PSMU. In contrast, those with high EI are more adept at recognizing and managing unpleasant emotions, which lowers psychological stress (23, 24). Previous studies have indicated that low trait EI may trigger problems related to internet addiction (25–27). Recent research has suggested that EI can protect against PSMU (28). Individuals who excel at understanding and regulating their emotions can better adapt to society and engage in face-to-face socialization, reducing their reliance on social media to maintain social connections or share activities. This reduced the risk of social media overuse (29). Higher levels of EI enable individuals to handle emotion-related problems more rationally, better control their emotions, understand others’ emotions, and use the Internet appropriately, thereby preventing Internet addiction (30). In summary, EI was associated with PSMU, with higher EI negatively predicting it.

### 1.2 Mediating role of social exclusion

The I-PACE model describes affective and cognitive processes as explanatory mechanisms for Internet-related disorders. Social exclusion is an affective factor, while experiential avoidance is a cognitive factor. Social exclusion occurs when an individual is excluded by a social group, which blocks their belonging and relationship needs and leads to feelings of neglect and loneliness. Social exclusion included neglect, rejection, isolation, and denial (31). A correlation may exist between EI and social exclusion. Individuals with low EI are more likely to social exclusion from friends and family, whereas those with high EI can improve their social competence, interpersonal relationships, and social support (32). Additionally, negative life events such as social exclusion are distal factors that trigger adolescent addiction to social media (33). According to the CIUT (20), socially excluded individuals seek psychological resources to satisfy emotional requirements unavailable in offline life. As a convenient compensation method, social media can alleviate negative emotions, such as feelings of exclusion and loneliness, perceived in real-life interpersonal interactions. However, prolonged and high-frequency social media use increases the risk of PSMU. According to general stress theory (34), social exclusion is a stressful event in the external environment that makes socially excluded individuals prone to deviant online behaviors (35). Thus, social exclusion may mediate the effects of EI on PSMU.

### 1.3 Mediating role of experiential avoidance

Based on the I-PACE model, experiential avoidance, a nonadaptive cognition, involves actively avoiding unpleasant thoughts and emotions and an inability to accept and experience one’s true thoughts and emotions well (36–38). Previous research indicated a significant association between emotional dysregulation and experiential avoidance. The ability to regulate emotions significantly predicts experiential avoidance. The lower an individual’s EI, the more likely they are to adopt avoidant behaviors when encountering difficulties. Experiential avoidance, a method of avoiding negative emotions and experiences, can predispose individuals to fall into an avoidance pattern by allowing them to escape threats for brief periods of pleasantness (39). Previous research indicates that PSMU is an anxiety avoidance strategy associated with experience avoidance (40). Highly socially avoidant adolescents who experience experiential avoidance when encountering unpleasant stimuli are prone to developing PSMU (41). Thus, experiential avoidance may mediate the effects of EI on PSMU.

It has been shown that the experience of social exclusion is closely related to experiential avoidance tendencies, and both significantly predict PSMU among college students (41–43). This may be due to cultural factors specific to Chinese college students, especially collectivist norms, which may increase college students’ sensitivity to social exclusion and avoidance. College students are more sensitive to group belonging, and social exclusion is more likely to trigger avoidant coping, thus seeking emotional regulation and virtual belonging through increased social media use (44). Therefore, social exclusion and experiential avoidance were chosen for this study to provide insight into the psychological mechanisms underlying PSMU.

### 1.4 Present study

Previous studies have explored the relationship between EI and PSMU, but most of them have focused on Western samples, and not enough relevant studies have been conducted for Chinese populations. There are not enough studies exploring the potential mediating mechanisms between EI and PSMU for Chinese samples. It is of great significance to conduct this study for this research area. On the one hand, it can expand the applicability of the existing results in different cultural contexts, and on the other hand, it can help to deepen the understanding of the occurrence mechanism of PSMU among Chinese college students. This study aimed to investigate how EI affects PSMU among Chinese college students and to provide some basic references for PSMU intervention.

**Figure 1:**
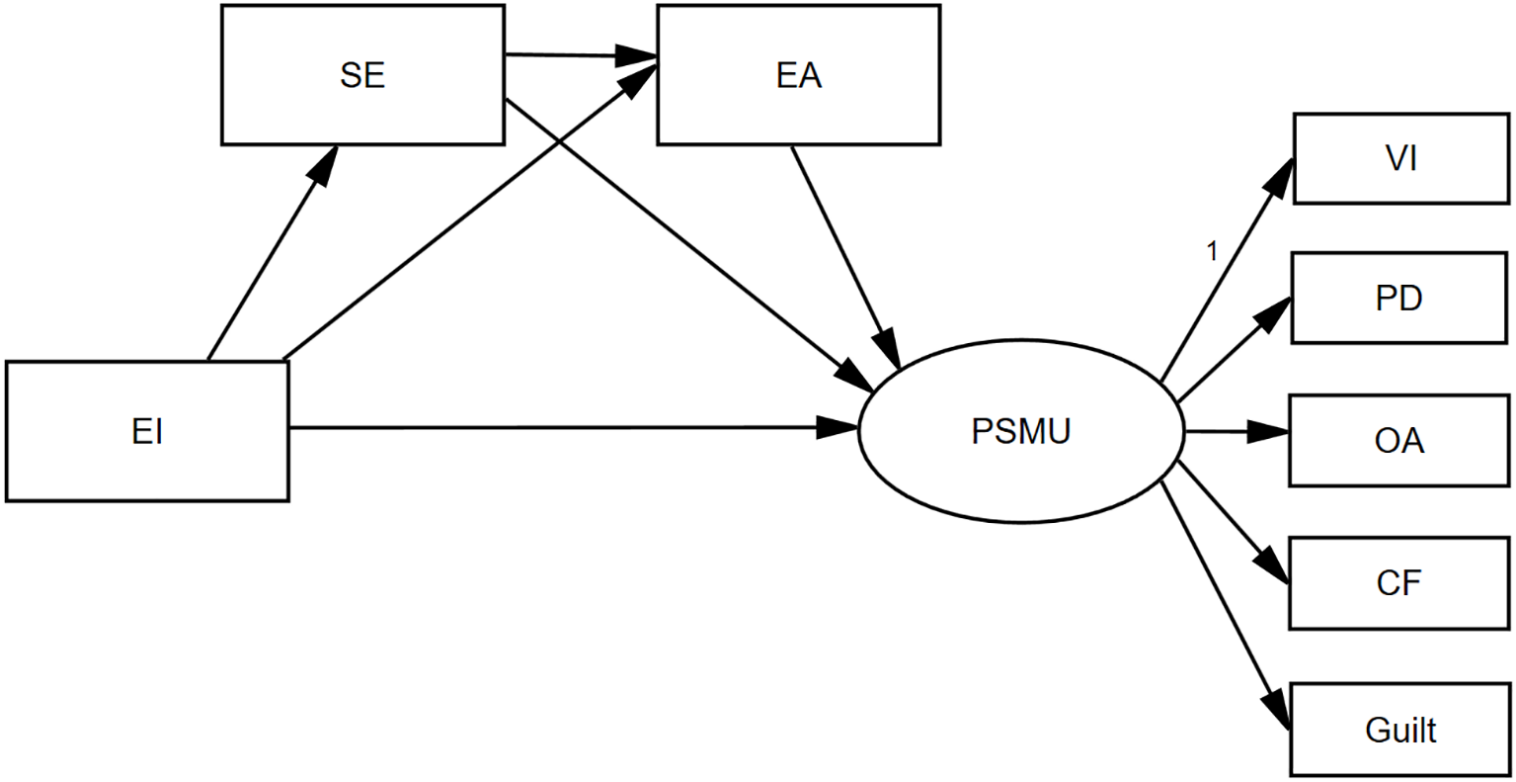
Theoretical model. EI, emotional intelligence; PSMU, problematic social media use; SE, social exclusion; EA, experiential avoidance; VI, viscosity increase; PD, physiological damage; OA, omission anxiety; CF, cognitive failure. The same as below.

Therefore, the I-PACE model served as a theoretical foundation, EI as a core personal trait, social exclusion and experiential avoidance as affective and cognitive factors, respectively, and PSMU as a pattern of problematic behavior. **Figure 1** illustrates the proposed model with the following assumptions:

H1: EI negatively correlates with social exclusion, experiential avoidance, and PSMU.
H2: EI indirectly affects PSMU through social exclusion (H2a) and experiential avoidance (H2b).
H3: The relationship between EI and PSMU is serially mediated by social exclusion and experiential avoidance.

## 2 Methods

### 2.1 Participants

Convenience sampling was used to randomly select classes in nine public colleges and universities in Chengdu, Beijing, Shanghai, and Yunnan from May 1, 2021, to October 28, 2021. The survey was conducted through a psychological organization. The target participants were undergraduates and master’s degree students. The investigators surveyed the participants for informed consent. An informed consent statement was incorporated into the distributed paper questionnaire in written form. In this study, the participants’ completion and submission of the questionnaire indicated informed consent and voluntary participation. The content of the informed consent statement in the questionnaire is as follows (**Supplementary File S1**): “If you confirm that you have understood the content and purpose of this research survey and agree to participate, please complete this questionnaire based on your true situation. If you refuse to participate, please do not complete or return the questionnaire.” Participant anonymity was guaranteed. There were 1,448 valid questionnaires. The mean age difference between males and females was statistically significant (p < 0.01).

This study was approved by the Ethics Review Committee of Chengdu Medical College (approval number: 2021NO.07) and strictly adhered to the guidelines outlined in the Declaration of Helsinki.

### 2.2 Measures

#### 2.2.1 Wong and Law’s emotional intelligence scale-C

This scale is a Chinese revision of the scale compiled by Wang (22) based on the work of Wong et al. (45), with 16 entries and 4 dimensions (self-emotional assessment, others’ emotional assessment, emotional adjustment, and emotional use). “Self-Emotional Assessment” consists of items 1-4, with two items as examples below: I have a good sense of why I have certain feelings most of the time, and I have a good understanding of my own emotions. “Emotional adjustment” consists of items 5-8, with two items as examples below: I am able to control my temper so that I can handle difficulties rationally, and I am quite capable of controlling my own emotions. “Emotional use” consists of items 9-12, with two items as examples below: I always tell myself I am a competent person, and I am a self-motivating person. “Others’ emotional assessment” consists of items 13-16, with two items as examples below: I always know my friends’ emotions from their behavior, and I am sensitive to the feelings and emotions of others. On a seven-point rating system, responses range from 1 (“Strongly Disagree”) to 7 (“Strongly Agree”). The overall scores ranged from 16 to 112. Higher overall scores represented a higher EI. The scale is suitable for measuring EI in individuals. The scale has been translated into Chinese and applied in Chinese-speaking cultures with a high degree of cultural adaptability. The scale has been confirmed to be applicable to a sample of Chinese college students by either prior literature or prior research (46–49). The Cronbach’s α for the scale in this study was 0.908.

#### 2.2.2 Social exclusion questionnaire for undergraduates

The scale was developed by Wu et al. (31). The scale was used to measure social exclusion among university students. The scale comprises 19 items on a 5-point scale ranging from 1 (“Never”) to 5 (“Always”). Higher total scores indicated that individuals perceived social exclusion as stronger. The Cronbach’s α for the scale in this study was 0.958.

#### 2.2.3 Acceptance and action questionnaire second edition

This scale was designed by Bond et al. (50) and adapted to Chinese by Cao et al. (36). This scale was used to measure the level of experiential avoidance among college students. It comprises seven items on a seven-point scale ranging from 1 (“never true”) to 7 (“always true”). The overall scores ranged from 7 to 49. Higher overall scores indicate higher levels of experiential avoidance. The Cronbach’s α for the scale in this study was 0.914.

#### 2.2.4 Problematic mobile social media usage assessment questionnaire for adolescents

This scale is a behavioral assessment questionnaire for PSMU among adolescents developed by Jiang (11). The scale can be used to measure the level of PSMU among adolescents or college students. It contains 20 items categorized into five factors: viscosity increase, physiological damage, omission anxiety, cognitive failure, and guilt. The scale uses a five-point scale (1 = “Not at All,” 5 = “Fully”). A higher overall score represented a more severe tendency toward PSMU. The Cronbach’s α for the scale in this study was 0.951.

### 2.3 Procedures

In all Chinese universities, there are regular psychological support groups consisting of psychology committee members. Each class has a psychological committee member. Professional psychology committee members were contacted through psychological support groups, and offline paper questionnaires were collected. Two graduate psychology students and one psychology committee member from each program collaborated to distribute and collect questionnaires. Two graduate psychology students informed the participants of the study’s purpose before distributing the questionnaires. The participants initiated the survey with their consent. Questionnaires were collected uniformly after distribution. The questionnaire comprised a consent section, guidelines, questions, and notes. A questionnaire was considered invalid if less than one-third of the questions were completed or if many responses showed a regular pattern. After removing 127 invalid questionnaires from the 1,575 recovered questionnaires, 1,448 valid questionnaires with a validity rate of 91.94% remained.

### 2.4 Data analysis

Data were analyzed using SPSS Windows software version 22.0, employing descriptive statistics, independent samples t-tests, ANOVA, and correlation analysis. Structural equation modeling was performed using AMOS 22.0, and the mediating effect was tested using SPSS-Process 3.3. Statistical significance was set at *p* < 0.05. The model fit indices were as follows: AGFI, GFI, NFI, CFI, and TLI > 0.95; RMSEA < 0.05 (good model); AGFI, GFI, NFI, CFI, and TLI > 0.80; RMSEA < 0.08 (acceptable models) (51, 52). Harman’s one-factor test was used to test Common Method Deviation, with the explanatory power of the first factor not surpassing the critical value of 50% (53).

## 3 Results

### 3.1 Common method deviation test

The first-factor interpretation percentage was 38.44%, lower than the critical value criterion of 50%, suggesting no serious common method bias (53).

### 3.2 Basic information and sex differences in emotional intelligence, social exclusion, experiential avoidance, and problematic social media use

**Table 1** presents the basic information and sex differences for each variable. After removing 127 invalid questionnaires from the 1,575 recovered questionnaires, 1,448 valid questionnaires with a validity rate of 91.94% remained. Of them, 732 (50.8%) were males and 708 (49.2%) were females. Their overall mean age was 20.94 ± 2.07, with 21.07 ± 2.18 years for males and 20.79 ± 1.94 years for females. Gender differences appeared in social exclusion, with males scoring significantly higher than females on social exclusion (*p* < 0.05). Other factors showed no significant sex-related differences.

**Table 1:** Basic information and sex differences across variables.

Variables	*M* ± *SD*	Male *M* ± *SD*	Female *M* ± *SD*	*t*
	*N* = 1448	*n* = 732	*n* = 708	
1. EI	75.5 ± 17.21	75.44 ± 17.07	75.46 ± 17.40	–0.02
2. Social exclusion	39.05 ± 16.64	40.11 ± 16.67	38.07 ± 16.60	2.32*
3. Experiential avoidance	24.72 ± 9.56	25.02 ± 9.49	24.44 ± 9.65	1.15
4. PSMU	61.71 ± 19.15	61.72 ± 19.35	61.82 ± 19.00	–0.1
Viscosity increase	17.15 ± 5.13	17.23 ± 5.13	17.10 ± 5.15	0.51
Physiological damage	14.78 ± 5.89	14.79 ± 5.92	14.82 ± 5.86	–0.10
Omission anxiety	12.45 ± 4.39	12.55 ± 4.43	12.37 ± 4.35	0.76
Cognitive failure	11.47 ± 3.99	11.33 ± 3.99	11.63 ± 4.00	–1.43
Guilt	5.85 ± 2.63	5.81 ± 2.70	5.90 ± 2.56	–0.62

* denotes *p* <.05.

### 3.3 Correlational analysis

**Table 2** shows that EI was substantially negatively correlated with social exclusion, experiential avoidance, and PSMU. Social exclusion was substantially positively correlated with experiential avoidance and PSMU. Experiential avoidance was substantially positively correlated with PSMU. Therefore, H1 was supported.

**Table 2:** Bivariate correlation analysis of variables.

Variables	Social exclusion	Experiential avoidance	PSMU	Viscosity increase	Physiological damage	Omission anxiety	Cognitive failure	Guilt
EI	–0.673**	–0.607**	–0.566**	–0.504**	–0.526**	–0.534**	–0.428**	–0.418**
Social exclusion	1	0.661**	0.567**	0.525**	0.532**	0.593**	0.376**	0.353**
Experiential avoidance	0.661**	1	0.619**	0.584**	0.574**	0.570**	0.453**	0.448**

** denotes *p* <.01.

### 3.4 Mediating effect test of the overall model

**Figure 2:**
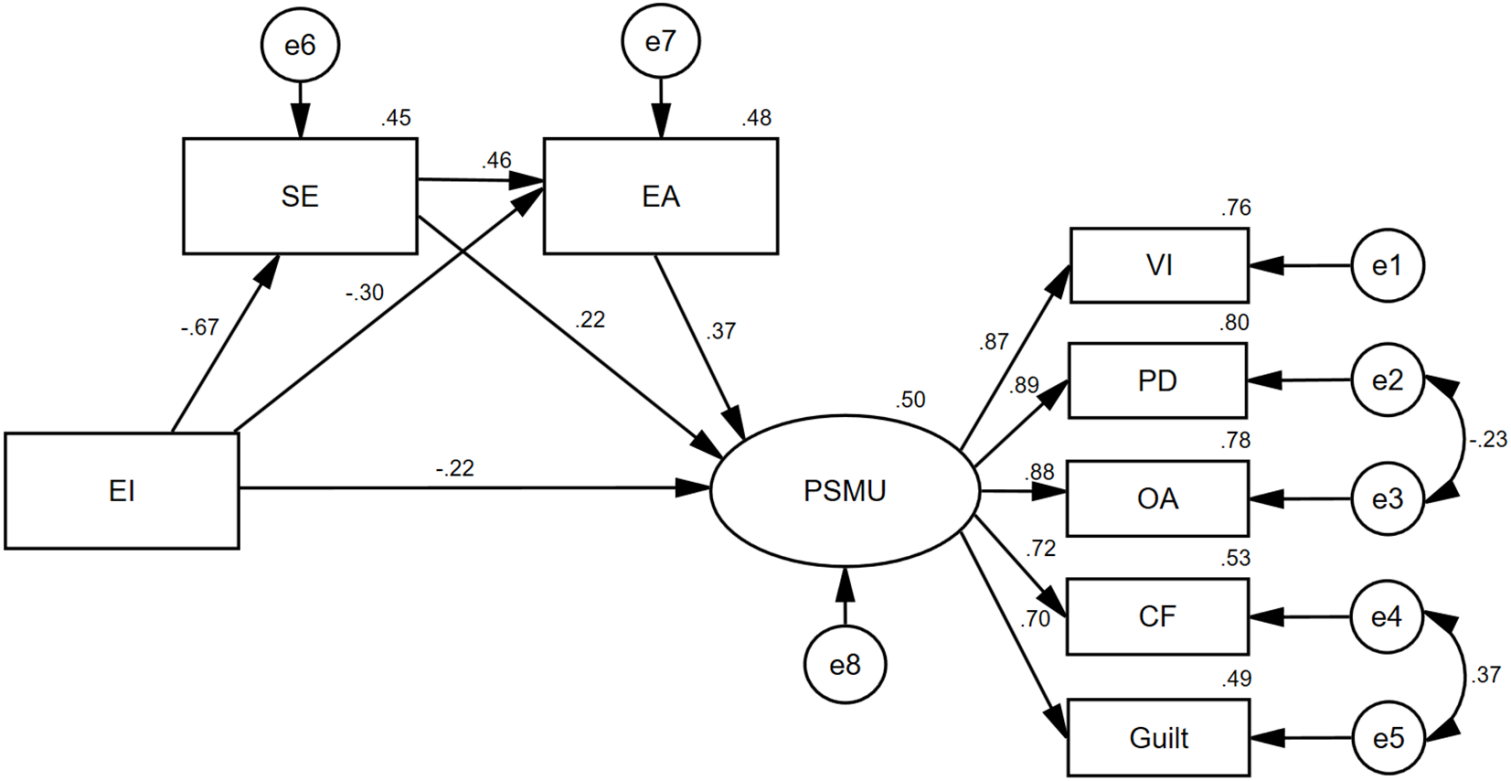
Mediating role of social exclusion and experiential avoidance on the impact of emotional intelligence on problematic social media use.

This study employed AMOS to analyze data from 1,448 college and university students. The results showed that All pathways were statistically significant (*p* < 0.05). Guided by the modification indices (MI), the initial model was revised to obtain a final model with a good fit (**Figure 2**). **Table 3** lists the model fit indices.

**Table 3:** Model fit comparison.

Model	*X2*	*df*	*X2/df*	AGFI	GFI	NFI	CFI	TLI	RMSEA
Initial model	298.905	17	17.583	0.888	0.947	0.962	0.964	0.940	0.107
Final model	111.257	15	7.417	0.954	0.981	0.986	0.988	0.977	0.067
Indices				> 0.95	> 0.95	> 0.95	> 0.95	> 0.95	< 0.08
				Good	Good	Good	Good	Good	Acceptable

The final model (**Figure 2**) demonstrated that EI directly predicted PSMU. Both social exclusion and experiential avoidance partially mediate the effects of EI on PSMU. Social exclusion and experiential avoidance sequentially mediated the impact of EI on PSMU.

This study employed the SPSS add-in process (54) and further tested mediating effects using the bias-corrected percentile bootstrap method (5,000 replicate samples). The results (**Table 4**) show that the 95% confidence intervals for all paths exclude zero, suggesting that all paths are significant.

**Table 4:** Bootstrap analysis of the mediated effects test.

Effect		Effect value	*SE*	95% CI	Ratio (%)
Total effect	EI→PSMU	–0.63	0.02	[–0.68, –0.58]	
Direct effects	EI→Social exclusion	–0.65	0.02	[–0.69, –0.61]	
	EI→Experiential avoidance	–0.16	0.01	[–0.19, –0.14]	
	Social exclusion→Experiential avoidance	0.27	0.02	[0.24, 0.29]	
	Social exclusion→PSMU	0.20	0.03	[0.13, 0.26]	
	Experiential avoidance→PSMU	0.74	0.05	[0.63, 0.85]	
	EI→PSMU	–0.25	0.03	[–0.31, –0.19]	39.68%
Indirect effects	EI→Social exclusion→PSMU	–0.13	0.02	[–0.17, –0.08]	20.63%
	EI→Experiential avoidance→PSMU	–0.12	0.02	[–0.16, –0.09]	19.05%
	EI→Social exclusion→Experiential avoidance→PSMU	–0.13	0.01	[–0.15, –0.10]	20.63%

## 4 Discussion

### 4.1 Current situation of college students’ emotional intelligence, social exclusion, experiential avoidance, and problematic social media use

This study demonstrated that no significant sex differences existed in any of the variables except for social exclusion, with males having higher social exclusion scores than females. This finding suggests that males were more socially excluded than females, which is consistent with the findings of Lev-Wiesel et al. (55), possibly because males are more impulsive, less able to control their emotions and behavior during interpersonal conflicts, more likely to conflict with others, and consequently, more likely to experience social exclusion over time. Being more affectionate and warm, females tended to develop similar traits. They believe they are more jeopardized by social exclusion than males, making them more reluctant to experience rejection (56). Previous studies have suggested sex differences in EI (57). Consistent with Marco et al. (58), our study failed to find any significant sex differences in EI. EI manifests in introspection, stress management, and interpersonal relationships. Males outperformed females in introspection and coping with stressful emotions. Females outperformed males in terms of empathy and socialization. While the dominant EI abilities differed between sexes, there was no absolute difference in the overall level of EI, explaining the lack of significant sex differences in EI scores. This study found no significant gender differences in experiential avoidance, consistent with the results of Munsamy et al. (59). This may be because both males and females experience emotional stress and anxiety when encountering difficulties, leading to certain avoidance behaviors and resulting in minimal sex-based differences in experiential avoidance. Additionally, there were no significant sex differences in PSMU, consistent with Primack et al. (60). This may be due to the continuous progress in modern information and communication technology, increasingly powerful smartphones, and social applications that align with individual needs. The widespread use of social applications, such as WeChat, TikTok, and QQ, has normalized this phenomenon in social network communication. Smartphones’ portability and ease of use make them less susceptible to external factors. Therefore, there is little distinction between males and females regarding the opportunities and duration of their social media use, nor is there much difference in the effects of their usage on themselves (61).

### 4.2 Impact of emotional intelligence on problematic social media use

This study aimed to investigate how EI affects PSMU. The results showed that EI was negatively related to PSMU and directly predicted it. EI acts as a core personal trait that protects against PSMU. College students with higher EI were at a lower risk of PSMU, consistent with the findings of Barberis et al. (28). PSMU can be considered a maladaptive and negative coping mechanism (62). College students with lower EI are less able to manage stress-related negative emotions, thus adopting nonadaptive strategies to cope with emotional problems, diverting attention, and alleviating distress through external resources, such as social media, ultimately resulting in PSMU (63, 64). According to impulsivity theory (65), individuals with high emotional control exhibit less impulsive Internet use. Therefore, improving the EI of college students can be an intervention for PSMU, leading to improved functioning in areas such as scholastic achievement, interpersonal interactions, and physical and psychological well-being (66). It can also reduce the tendency toward addictive behaviors (30).

### 4.3 Mediating role of social exclusion in the effect of emotional intelligence on problematic social media use

EI was negatively correlated with social exclusion, whereas social exclusion was positively correlated with PSMU. EI indirectly influences PSMU through social exclusion, supporting H2a. Prior studies have examined how EI affects peer interactions and social support (67). However, there is not enough literature on the direct effects of EI on social exclusion in Chinese samples. This study confirmed that EI affects social exclusion. Individuals with high EI are more adept at perceiving and utilizing social support (67). Shuo et al. found that EI was associated with social support. Individuals with higher EI are likely to receive more social support (68). The results of the structural equation modeling analysis in this study suggest that EI influences social exclusion with a path coefficient of -0.67, and social exclusion influences PSMU with a path coefficient of 0.22. The results of the mediation effect test showed that the mediation effect size for social exclusion was -0.13. This suggests that individuals with higher levels of EI tend to feel lower levels of social exclusion and thus their risk of PSMU is relatively low. They can identify, perceive, understand, and experience emotions (both their own and those of others) more effectively in everyday interpersonal interactions. Consequently, they are more socialized, more likely to develop positive attitudes, and respond with appropriate emotions and behaviors, resulting in greater social support from family, friends, and other contacts. This process improves mental health, provides social support and resources, and reduces indirect exclusion (68). Individuals with high EI are adept at managing and utilizing their emotions and cultivating positive emotional states through social interactions. This emotion affects those around them, promoting real-life interactions and reducing online socialization, consistent with the connotations of emotional contagion (69, 70). Furthermore, they understood how to effectively utilize social resources when encountering difficult situations, actively participate in social interactions, and reduce social alienation. This may reduce their need for access to psychological support and help via the Internet, leading to less time spent on social networks and thereby avoiding PSMU (71, 72). Conversely, college students with low EI struggle to control their emotions, which often leads to interpersonal conflict and social exclusion, particularly direct exclusion (73). To mitigate the unpleasant experiences triggered by social exclusion, they often take measures to avoid negative situations and alleviate negative emotions (74). Social media offer a safe environment for these students to share photos, express interest, interact with their peers, and build close online friendships (75), helping them avoid the interpersonal pressures of reality and negative emotions caused by social exclusion. Over time, these behaviors can lead to PSMU. Additionally, these behaviors gradually form a conditioned reflex, leading to the failure of self-control after continuous reinforcement, thus increasing addiction to social media and exacerbating their PSMU (76).

### 4.4 Mediating role of experiential avoidance in the effect of emotional intelligence on problematic social media use

There was a negative correlation between EI and experiential avoidance and a positive correlation between experiential avoidance and PSMU. EI indirectly influences PSMU through experiential avoidance, supporting H2b. MacCann et al. (77) found that emotion management skills were associated with avoidant coping. Individuals with poorer emotion management had more corresponding avoidance behaviors. The results of the structural equation modeling analysis in this study showed that EI had an effect on experiential avoidance with a path coefficient of -0.30, and experiential avoidance had an effect on PSMU with a path coefficient of 0.37. The results of the mediation effect test showed that the mediation effect size for experiential avoidance was -0.12. This suggests that individuals with higher EI tend to have lower levels of experiential avoidance, and thus their risk of PSMU is relatively low.

College students with higher EI were better able to manage emotions, regulate the external expression of emotions, and improve their internal psychological adaptation levels to resolve anxiety and fear when faced with pressure, unpleasant emotional states, and psychological changes. Therefore, when faced with stress, college students with high EI do not avoid or treat it negatively but adopt an accepting attitude toward facing the problem, enhancing their psychological development and reducing the likelihood of using experiential avoidance. This is consistent with Aljarboa et al. (78), who emphasized that college students with higher EI are less likely to exhibit avoidance behaviors (78). If an individual employs inappropriate emotion-regulation strategies, it may lead to the use of coping mechanisms for vicarious avoidance. Experiential avoidance can quickly alleviate the negative experience of an undesirable situation in the short term (39) and may be helpful and adaptive when dealing with negative emotions. However, this can be harmful if it becomes habitual, such as using social media as compensation. College students use social media to escape real life and negative emotions and to compensate for realistic frustrations and a lack of interpersonal responses through online socialization. When this behavior becomes habitual, it can lead to PSMU, resulting in social media addiction, consistent with the findings of Garcia-Oliva and Piqueras (79). Quan proposed a new perspective on addiction based on the connotation of experiential avoidance: the self-centered experiential avoidance model (80). This model explains addiction as a strategy for experiential avoidance and as a way for individuals with an addiction to interact with the social dimension. This model states that experiential avoidance can flexibly modulate an individual’s addiction level, which is consistent with the findings of the present study.

### 4.5 Chain mediating roles of social exclusion and experiential avoidance in the effect of emotional intelligence on problematic social media use

Based on previous research (81), this study suggests that social exclusion and experiential avoidance mediate the effects of EI on PSMU. According to Bennett (81), social exclusion positively predicts experiential avoidance, suggesting chain mediation. Compared to the direct impact of EI on PSMU, the chain-mediated effect was smaller but significant. The chain-mediating role was comparable to the separate mediating roles of social exclusion and experiential avoidance and was stable in the model. The chain-mediated model used in this study supports the interaction theory of addictive behaviors proposed by the I-PACE model, which explains affective and cognitive processes as mechanisms of Internet-related disorders. EI as a core personal trait, social exclusion as an affective factor, experiential avoidance as a cognitive factor, and PSMU as a pattern of problematic behavior. College students with lower EI were less able to recognize, assess, control, and apply their emotions. Consequently, they are less likely to comprehend others’ feelings and may be prone to conflict when their emotions are unstable. This makes them more susceptible to social exclusion, leading to loneliness and loss, which encourages them to avoid social contact and develop avoidance behaviors. Once accustomed to this avoidance pattern, they fall into experiential avoidance as the first sign of frustration or rejection (41, 44), using social media as a substitute for meeting social needs in real life. This behavior eventually leads to PSMU and a potential addiction to social media. Thus, social exclusion and experiential avoidance mediate the effect of EI on PSMU, supporting H3.

The hypotheses and the theoretical model of this study (**Figure 1**) were validated by the results of the study. EI negatively correlates with social exclusion, experiential avoidance, and PSMU. EI indirectly affects PSMU through social exclusion (H2a) and experiential avoidance (H2b). The relationship between EI and PSMU is serially mediated by social exclusion and experiential avoidance.

This study provides an empirical basis for the I-PACE model and highlights that core personal characteristics, combined with affective and cognitive factors, may trigger specific problematic Internet use behaviors. EI positively predicts PSMU. Social exclusion and experiential avoidance partially mediate the effect of EI on PSMU. These two variables also chain-mediate the impact of EI on PSMU. PSMU is the result of the interaction of personality traits, affective responses, and cognitive processes. Based on this, the present study proposes that college students with lower EI are more likely to experience a sense of social exclusion in group life due to their limited ability to perceive and regulate emotions, which in turn stimulates the tendency of experiential avoidance to alleviate emotional distress by avoiding internal pain. Online social media has become an important way for individuals to vicariously fulfill their social needs due to its instant gratification and low-risk interaction properties, but long-term use predisposes them to PSMU. This pathway reveals the mechanisms by which personality traits (EI) influence PSMU through affective responses (feelings of social exclusion) and cognitive responses (experiential avoidance), enriching the I-PACE model’s understanding of the mechanisms by which problematic Internet use behaviors occur.

In the future, educators should focus on developing EI in college students. Methods such as campus activities, themed class meetings, psychology classes, psychological counseling, and group counseling can help college students recognize and manage their emotions when they encounter difficulties, cultivate positive interpersonal relationships, and perceive the support of others, thereby reducing social exclusion. Consequently, college students may be less likely to engage in avoidance behaviors, effectively preventing PSMU. In future research, targeting internet addiction among college students, especially Reducing PSMU and improving mental health among college students can begin with increasing EI and reducing social exclusion and experiential avoidance among college students.

### 4.6 Limitations and future directions

This study has some limitations. First, although the participants were widely distributed, with 1,448 students enrolled in nine colleges and universities in Chengdu, Beijing, Shanghai, and Kunming, all were college students; thus, the sample was relatively homogeneous. Further research is necessary to determine whether the mechanisms of action of the study variables can be extrapolated to other groups. Future research should use samples from other age groups to address sample bias. Second, the questionnaires were administered independently. Participants may have been affected by the social approval effect when completing the questionnaire. Clinical assessments or event sampling should be used in future studies to gather information, as this will significantly reduce retrospective bias and enhance ecological validity. Third, In addition, the correlational nature of this study should be acknowledged as a limitation. Although significant associations were found among the variables, the cross-sectional and non-experimental design does not allow for causal inferences. Future research using longitudinal or experimental methods is needed to further clarify the factors that influence and shape PSMU mechanisms.

## 5 Conclusion

This study provides an empirical basis for the I-PACE model and highlights that core personal characteristics, in combination with affective and cognitive factors, may trigger specific problematic Internet use behaviors. The conclusions of this study are as follows: First, EI directly predicted PSMU. Second, social exclusion mediates the effects of EI on PSMU. Third, experiential avoidance mediated the impact of EI on PSMU. Finally, the social exclusion and experiential avoidance chains mediated the effect of EI on PSMU.

## Data Availability

The original contributions presented in the study are included in the article/**Supplementary Material**. Further inquiries can be directed to the corresponding authors.

## Supplementary material

Supplementary File S1 Research Questionnaire. (PDF).

Supplementary File S2 Raw Data. (EXCEL).
