# Supplementary material for: Effect of emotional intelligence on problematic social media use among Chinese college students: mediating role of social exclusion and experiential avoidance
Source: Front Psychiatry. 2025 Jun 3;16:1567060. doi: 10.3389/fpsyt.2025.1567060 (PMC12172549; doi:10.3389/fpsyt.2025.1567060)
Supplement: Supplementary File S1 — Research Questionnaire. (PDF). [file DataSheet1.pdf]

Last four digits of student number:_____________

Hello! First of all, thank you for participating in my survey! Second, your results are only for research and absolutely confidential and do not need to fill in your name. Please fill in according to your own true situation (type "√" only). This questionnaire surveys the use of the network and related influencing factors, please answer each one, do not miss items. There are no right or wrong options, and there is no value judgment involved. The mobile communication devices mainly used in the "social networks" mentioned in this survey refer to mobile phones, tablets and computers. The above contains instructions for informed consent. Your answer to the questionnaire indicates that you have given informed consent. If you confirm that you have understood the content and purpose of this research survey and agree to participate, please complete this questionnaire based on your true situation. If you refuse to participate, please do not complete the questionnaire and return it.

1. Gender: ① male ② female **2. Age:__________**

3. The only child: ① yes ② no **4. your major:__________**

5. Grade: ① freshman ② sophomore ③ junior ④ senior (senior five) ⑤ graduate student

1 What kind of social network do you use more?

A. Instant messaging category (such as WeChat, QQ, etc.)

B. Non-instant messaging category (Weibo, Xiaohongshu, TikTok, etc.)

C. both

2 In addition to work and study, you will use the Internet to socialize for ( ) hours per day.

3. What kind of people do you communicate more with when you are using social networks?

A. People you know in reality B. strangers on the Internet C. both

Please evaluate and judge your own situation according to the classification in the item column according to your own actual feelings and experience, and mark " √ " on the most consistent number.

**title**	**Strongly Disagree**	**Disagree**	**Slightly Disagree**	**Neutral**	**Slightly Agree**	**Agree**	**Strongly Agree**
1. I have a good sense of why I have certain feelings most of the time.	1	2	3	4	5	6	7
2. I have good understanding of my own emotions.	1	2	3	4	5	6	7
3. I really understand what I feel.	1	2	3	4	5	6	7
4. I always know whether or not I am happy.	1	2	3	4	5	6	7
5. I am able to control my temper and handle difficulties rationally.	1	2	3	4	5	6	7
6. I am quite capable of controlling my own emotions.	1	2	3	4	5	6	7
7. I can always calm down quickly when I am very angry.	1	2	3	4	5	6	7
8. I have good control of my own emotions.	1	2	3	4	5	6	7
9. I always set goals for myself and then try my best to achieve them.	1	2	3	4	5	6	7
10. I always tell myself I am a competent person.	1	2	3	4	5	6	7
11. I am a self-motivated person.	1	2	3	4	5	6	7
12. I would always encourage myself to try my best.	1	2	3	4	5	6	7
13. I always know my friends emotions from their behaviour.	1	2	3	4	5	6	7
14. I am a good observer of others emotions.	1	2	3	4	5	6	7
15. I am sensitive to the feelings and emotions of others.	1	2	3	4	5	6	7
16. I have good understanding of the emotions of people around me.	1	2	3	4	5	6	7

title	Not at All	Slightly	Moderately	Very	Fully
1.Every day, I unconsciously and frequently flip through the phone app, check the circle of friends dynamic, etc., and I can not remember how many times.	**1**	**2**	**3**	**4**	**5**
2. I always unconsciously pick up my mobile phone to open the social APP and flip through it aimlessly.	**1**	**2**	**3**	**4**	**5**
3. I always inadvertently prolong the use of mobile social networks on cell phones without realizing it.	**1**	**2**	**3**	**4**	**5**
4. I spend a lot of time every day logging in and checking Moments.	**1**	**2**	**3**	**4**	**5**
5. I have developed a dependence on mobile social networks on my cell phone, and sometimes I can't control the amount of time I spend playing.	**1**	**2**	**3**	**4**	**5**
6. Frequent and long-term use of mobile social networks to browse Moments and information often makes my eyes dry and visually tired.	**1**	**2**	**3**	**4**	**5**
7. I use my cell phone for a long time to brush my WeChat Moments, chat and browse information, and maintain a fixed posture, which often makes my cervical spine sore.	**1**	**2**	**3**	**4**	**5**
8. Using my fingers to slide the phone screen for a long time often makes my fingers muscles sore.	**1**	**2**	**3**	**4**	**5**
9. My prolonged use of mobile social networking swipes has caused my eyesight to deteriorate significantly.	**1**	**2**	**3**	**4**	**5**
10. Frequent and prolonged use of mobile social networks often leads to sleep deprivation and poor sleep quality.	**1**	**2**	**3**	**4**	**5**
11. I get worried and anxious when my phone suddenly doesn't connect to the internet and I can't check my social apps.	**1**	**2**	**3**	**4**	**5**
12. If you don't check WeChat or Weibo on your mobile phone for a while, you will always worry about missing or missing any information.	**1**	**2**	**3**	**4**	**5**
13. As long as I open the mobile social APP, I don't want to quit. I always want to see it for a while before I am satisfied.	**1**	**2**	**3**	**4**	**5**
14. I always want to control the time, frequency, and intensity of using mobile social networks, but it always doesn't work.	**1**	**2**	**3**	**4**	**5**
15. Due to the sheer volume of information on mobile social networks and their fast updates, I don't have time to consider the value of this information.	**1**	**2**	**3**	**4**	**5**
16. Use frequent and long time to watch moments, and less time to think deeply than before.	**1**	**2**	**3**	**4**	**5**
17. Due to the convenience of mobile phone networks, I rarely use my brain to remember, leading to my worse and worse memory.	**1**	**2**	**3**	**4**	**5**
18. Frequent and prolonged cell phone use of mobile social networks reduces communication with real-life friends and family.	**1**	**2**	**3**	**4**	**5**
19. I often feel regret and guilt when I use mobile social networks to swipe or chat for too long to delay my studies or work.	**1**	**2**	**3**	**4**	**5**
20. When I'm delayed in doing my business because I'm using social networks, I often regret the time I've lost by playing with my phone.	**1**	**2**	**3**	**4**	**5**

title	Never true	Very seldom true	Seldom true	Sometimes true	Frequently true	Almost always true	Always true
1. My painful experiences and memories make it difficult for me to live a life that I would value.	1	2	3	4	5	6	7
2. I'm afraid of my feelings.	1	2	3	4	5	6	7
3. I worry about not being able to control my worries and feelings.	1	2	3	4	5	6	7
4. My painful memories prevent me from having a fulfilling life.	1	2	3	4	5	6	7
5. Emotions cause problems in my life.	1	2	3	4	5	6	7
6. It seems like most people are handling their lives better than I am.	1	2	3	4	5	6	7
7. Worries get in the way of my success.	1	2	3	4	5	6	7

title	Never	Rarely	Sometimes	Often	Always
1. When people are flirting or playing with each other, people avoid me intentionally or not.	1	2	3	4	5
2. I became the object of a malicious tease.	1	2	3	4	5
3. Others will not share their moods or exchange experiences with me.	1	2	3	4	5
4. Others say bad things about me behind my back, affecting what others think of me.	1	2	3	4	5
5. My mistake was heckled or unceremoniously criticized.	1	2	3	4	5
6. I am often caught by others in mistakes, and omissions, and tattled by others.	1	2	3	4	5
7. When people are talking, I make the scene cold when I join in.	1	2	3	4	5
8. When I may make a fool of myself or make a mistake, others will only wait to see the joke.	1	2	3	4	5
9. Others would laugh at my shortcomings and sting me.	1	2	3	4	5
10. I would receive unkind looks for no reason.	1	2	3	4	5
11. When I am lost, I can not get advice or comfort from others.	1	2	3	4	5
12. Even if I had tried to improve the relationship, I didn't get a positive response.	1	2	3	4	5
13. Even if we know each other, others will not take the initiative to greet me.	1	2	3	4	5
14. Others are impatient and perfunctory with my inquiries or requests.	1	2	3	4	5
15. I had trouble getting an enthusiastic response to my proactive conversation.	1	2	3	4	5
16. Other people rarely notice me and don't know much about me.	1	2	3	4	5
17. Others will intentionally or unintentionally distance themselves from me in space.	1	2	3	4	5
18. When I talk to others, they don't answer me much, no matter what topic I'm talking about.	1	2	3	4	5
19. I seem to have trouble getting deeper or longer interactions with others.	1	2	3	4	5

Thank you for participating!

学号后四位：________________

您好！首先感谢您参加我的调查！其次，您的结果只用于研究并绝对保密，不需要填写您的姓名。请根据您自己的真实情况填写（打“√”即可）。本问卷调查网络使用情况与相关影响因素，请对每一条都进行作答，不要漏项。选项没有对错之分，也不涉及任何价值评判。本调查所说“社交网络”主要使用的移动通讯设备是指手机、平板与电脑等。上述内容包含知情同意的说明。您对问卷的回答表明您已知情同意。如果您确认已了解本次研究调查的内容和目的，并同意参与，请根据您的真实情况填写此问卷。如果您拒绝参与，请不要填写问卷并将其交回。

1、性别：①男 ②女 **2、年龄：**________________

3、您是否独生子女：①是 ②否 **4、您的专业：**________________

5、您的年级：①大一 ②大二 ③大三 ④大四（大五） ⑤研究生

1 您更多使用哪一类社交网络？

A、即时通讯类（如微信、QQ 等）

B、非即时通讯类（微博、小红书、抖音等）

C、两者皆有

2 您每天除工作学习外，使用网络进行社交的时间为（ ）小时。

3 在使用社交网络时，您更多跟什么样的人交流？

A、现实中认识的人 B、网络上的陌生人 C、两者皆有

请您根据自己的实际感受和体会，用下面的描述根据题项栏中的分级对您自身情况进行评价和判断，并在最符合的数字上划“√”。

题目	非常不符合	比较不符合	有点不符合	不能确定	有点符合	比较符合	非常符合
1.通常我都能知道自己产生某些感受的原因。	1	2	3	4	5	6	7
2.我很了解自己的情绪。	1	2	3	4	5	6	7
3.我真的能明白自己的感受。	1	2	3	4	5	6	7
4.我常常知道自己为什么觉得开心或不高兴。	1	2	3	4	5	6	7
5.遇到困难时，我能控制自己的脾气。	1	2	3	4	5	6	7
6.我很能控制自己的情绪。	1	2	3	4	5	6	7
7.当我愤怒时，我通常能在很短的时间内冷静下来。	1	2	3	4	5	6	7
8.我对自己的情绪有很强的控制能力。	1	2	3	4	5	6	7
9.我通常能为自己制定目标并尽量完成这些目标。	1	2	3	4	5	6	7
10.我经常告诉自己是一个有能力的人。	1	2	3	4	5	6	7
11.我是一个能鼓励自己的人。	1	2	3	4	5	6	7
12.我经常鼓励自己要做到最好。	1	2	3	4	5	6	7
13.我通常能从朋友的行为中猜到他们的情绪。	1	2	3	4	5	6	7
14.我观察别人情绪的能力很强。	1	2	3	4	5	6	7
15.我能很敏锐地洞悉别人的感受和情绪。	1	2	3	4	5	6	7
16.我很了解身边的人的情绪。	1	2	3	4	5	6	7

题目	完全不符合	不太符合	不清楚	比较符合	完全符合
1.每天都会无意识地频繁翻阅手机 APP、查看朋友圈动态等，自己都记不清有多少次。	1	2	3	4	5
2.我总是无意识地拿起手机打开社交 APP，漫无目的的随便翻看。	1	2	3	4	5
3.总是不经意间延长了使用手机移动社交网络的时间而没有觉察。	1	2	3	4	5
4.我每天都会花费大量的时间用来登录和查看朋友圈。	1	2	3	4	5
5.我对手机移动社交网络产生了一定的依赖，有时不能控制玩的时间。	1	2	3	4	5
6.频繁和长时间使用移动社交网络刷朋友圈和浏览信息，常使我的眼睛干涩、视觉疲劳。	1	2	3	4	5
7.长时间使用手机刷朋友圈、聊天和浏览信息，保持固定姿势，常使我的颈椎酸痛。	1	2	3	4	5
8.长时间使用手指滑手机屏幕，常使我的手指肌肉酸痛。	1	2	3	4	5
9.长时间使用手机移动社交网络刷屏，使我的视力明显下降。	1	2	3	4	5
10.频繁和长时间使用移动社交网络，常导致睡眠不足和睡眠质量差。	1	2	3	4	5
11.当手机突然联不上网，无法查看社交 APP 时，会感到担心和焦虑。	1	2	3	4	5
12.如果一会儿没有在手机上查看微信、微博等，总担心会遗漏或错过什么信息。	1	2	3	4	5
13.我只要打开手机社交 APP 就不愿意退出来,总想再看一会才会满足。	1	2	3	4	5
14.我总是想要控制使用手机移动社交网络刷屏的时间、频率和强度，但却总是没什么效果。	1	2	3	4	5
15.由于移动社交网络中的信息数量大、更新快，我没有时间去深入思考这些信息的价值。	1	2	3	4	5
16.频繁和长时间使用移动社交网络刷屏看朋友圈等，深入思考问题的时间比以前少了。	1	2	3	4	5
17.由于手机移动网络的便利，使我很少用脑子去记忆，这也导致了我的记性越来越不好。	1	2	3	4	5
18.频繁和长时间手机使用移动社交网络，与现实生活中朋友、家人沟通减少了。	1	2	3	4	5
19.当使用移动社交网络刷屏或聊天时间过长而耽误学习或工作，常会感到后悔和内疚。	1	2	3	4	5
20.因使用社交网络而耽误了做正事，常会感到后悔玩手机耽误了时间。	1	2	3	4	5

题目	从未	罕见	少见	有时	多见	常见	总是
1.痛苦的经历使我难以过上理想的生活。	**1**	**2**	**3**	**4**	**5**	**6**	**7**
2.某些感受让我感到害怕。	**1**	**2**	**3**	**4**	**5**	**6**	**7**
3.我担心控制不了自己的忧虑和感受。	**1**	**2**	**3**	**4**	**5**	**6**	**7**
4.痛苦的记忆破坏了我愉快的生活。	**1**	**2**	**3**	**4**	**5**	**6**	**7**
5.情绪扰乱了我的生活。	**1**	**2**	**3**	**4**	**5**	**6**	**7**
6.大多数人似乎比我更能应对生活。	**1**	**2**	**3**	**4**	**5**	**6**	**7**
7.瞻前顾后阻碍了我的成功。	**1**	**2**	**3**	**4**	**5**	**6**	**7**

题目	从不	偶尔	有时	经常	总是
1、大家相互调侃或打闹时有意无意避开我。	1	2	3	4	5
2、我成为被人恶意捉弄的对象。	1	2	3	4	5
3、大家不会与我分享心情或交流经验。	1	2	3	4	5
4、别人在背后说我的坏话，影响其他人对我的看法。	1	2	3	4	5
5、我的失误被起哄或毫不客气的批评。	1	2	3	4	5
6、我常被人抓住错处、疏漏和打小报告。	1	2	3	4	5
7、大家一起聊天时，我一加入进去就冷场。	1	2	3	4	5
8、在我可能出丑或出差错时，别人只会等着看笑话。	1	2	3	4	5
9、别人会嘲笑我的短处，刺痛我。	1	2	3	4	5
10、我会无缘无故的接收到非善意的眼神。	1	2	3	4	5
11、我失落时，得不到别人的劝解或安慰。	1	2	3	4	5
12、即便我已经努力改善关系，也得不到积极的回应。	1	2	3	4	5
13、即便彼此认识，别人也不会主动同我打招呼。	1	2	3	4	5
14、别人对于我的询问或请求满不耐烦，态度敷衍。	1	2	3	4	5
15、我的主动攀谈难以得到热情的回应。	1	2	3	4	5
16、别人很少会注意到我，也不太清楚我的情况。	1	2	3	4	5
17、别人会有意无意在空间上拉开与我的距离。	1	2	3	4	5
18、聊天时，不论我说什么话题，别人都不怎么接话。	1	2	3	4	5
19、我与别人的交流似乎难以深入或延长。	1	2	3	4	5

谢谢参与！
